# Superior mesenteric artery aneurysms in pediatric patients with congenital anomalies

**DOI:** 10.1016/j.jvscit.2025.102008

**Published:** 2025-10-08

**Authors:** Anush Motaganahalli, Muskaan Ramchandani, Mackenzie Madison, John Maijub, Ashley Gutwein, Alok Gupta

**Affiliations:** a Indiana University School of Medicine, Indianapolis, IN; b Division of Vascular Surgery, Indiana School of Medicine, Indianapolis, IN; c Division of Vascular Surgery, University of Colorado, Loveland, CO

**Keywords:** Aneurysm, Congenital, Pediatric, Vascular repair, Visceral artery

## Abstract

This is a case series of two pediatric patients with symptomatic superior mesenteric artery aneurysms. Visceral artery aneurysms are rare with an incidence of 0.1% to 2% in the general population. Pediatric visceral arterial aneurysms are even more rare. This report describes two unique cases of symptomatic pediatric superior mesenteric artery aneurysms. Both patients had congenital anomalies highlighting a potential syndromic association. The patients received autogenous conduits, which were grafted using an interrupted end-to-end technique. We believe that these grafts provide increased durability and a better fit, and that they should be considered as the conduit of choice for pediatric reconstructions.

Visceral artery aneurysms (VAAs) are a rare vascular pathology with aneurysms of the superior mesenteric artery (SMA) representing 6% to 15% of all VAAs. This condition is rare in the general population, and even more so in pediatric patients.^1^ Consequently, this is an understudied and highly vulnerable population for which there is limited literature pertaining to VAA management and outcomes among the pediatric population. This case report discusses the management of SMA aneurysms in pediatric populations, and the use of autogenous conduits grafted with an end-to-end technique as a tool for meaningful intervention.

## Case report

A 16-year-old female with Down syndrome and repaired congenital heart defects presented with abdominal pain, nausea, vomiting, and low-grade fevers. Laboratory studies showed a normal white-cell count and mildly elevated C-reactive protein; abdominal ultrasound was normal. On account of persistent, nonspecific gastrointestinal symptoms an abdominal computed tomography scan (CT) demonstrated a saccular distal 2.6 cm SMA aneurysm with mild perianeurysmal fat stranding. However, this was not considered as an infected aneurysm. The patient also had radiological findings consistent with cystitis. Proximal SMA was 5 mm and appeared healthy. Urinalysis grew group B streptococcus; blood cultures and echocardiography were negative for infective endocarditis.

**Fig 1:**
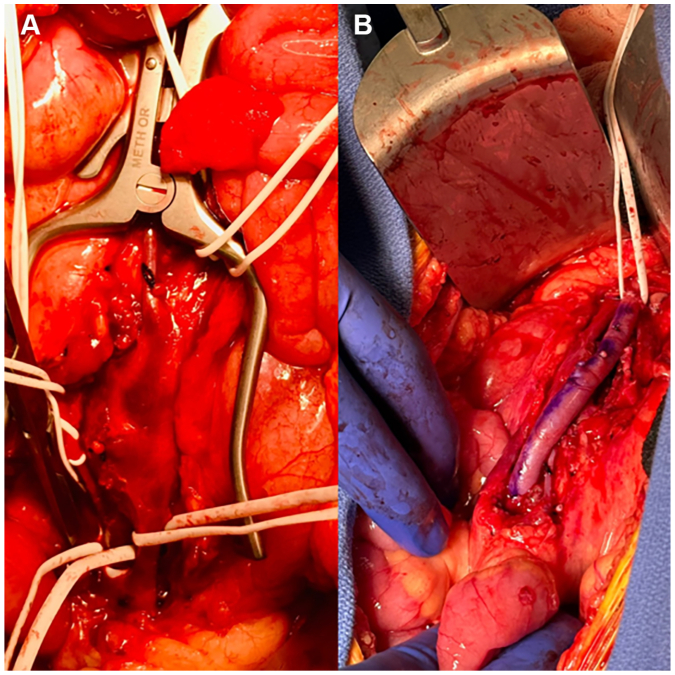
Shows intact superior mesenteric artery (SMA) aneurysm **(A)** and after reconstruction with saphenous vein graft **(B)**.

Due to the aneurysm’s large size and inflammatory rind, open repair with resection of the aneurysm and reversed great saphenous vein interposition was performed. A midline laparotomy was performed to gain access to the peritoneum. The proximal SMA was then dissected, followed by the distal portion of the SMA. Dense adherence to the superior mesenteric vein necessitated partial preservation of the posterior aneurysm wall (Fig 1, *A*); the remaining sac was excised, and an end-to-end interrupted anastomosis was completed (Fig 1, *B*). The branches of the SMA were controlled and preserved using vessel loops. A planned second-look laparotomy the next day confirmed viable bowel, and the abdomen was closed. Antibiotics to treat group B strep were stopped after 5 days per infectious disease recommendations. She was discharged to home on postoperative day 5 with no complications.

**Fig 2:**
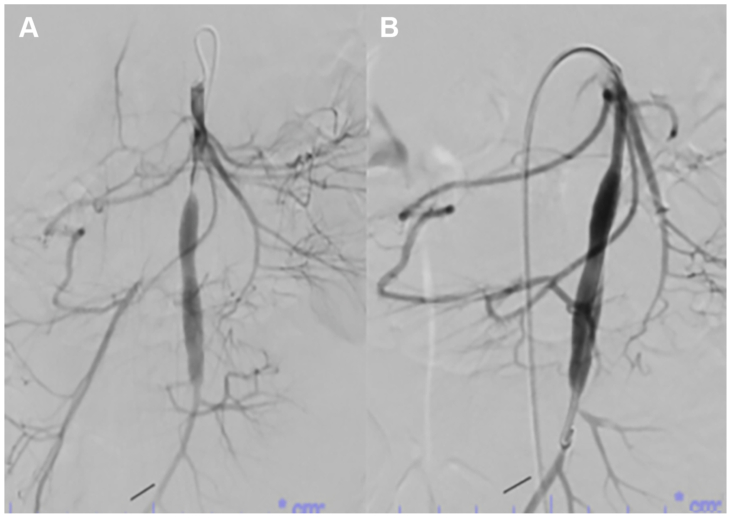
Shows proximal anastomotic stenosis of the vein graft **(A)** and results after successful angioplasty **(B)**.

One-month follow up CT angiography and duplex showed a wide patent bypass, and the patient’s symptoms had completely resolved. Two months later she represented with abdominal pain, vomiting, and diarrhea; CT angiography confirmed a high-grade proximal anastomotic stenosis (Fig 2, *A*) that was treated with a 5 × 2 mm balloon angioplasty. There was no residual stenosis after angioplasty (Fig 2, *B*). Recurrent stenosis at 3 months required placement of a 5 mm × 19 mm uncovered balloon-expandable stent in the proximal SMA. At 2-year follow-up, the patient was maintained on dual antiplatelet therapy with aspirin and clopidogrel. There was no recurrent stenosis noted on mesenteric duplex exam.

**Fig 3:**
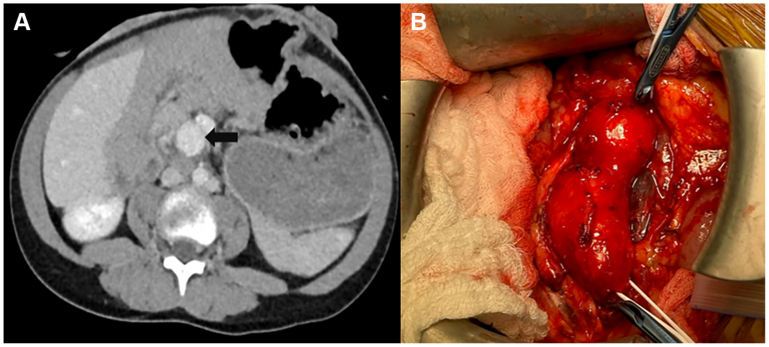
Shows a cross sectional computed tomography (CT) image, demonstrating a large superior mesenteric artery aneurysm (*black arrow*) **(A)** and intraoperative image of the aneurysm **(B)**.

The second patient is a 13-year-old boy with cloacal exstrophy who presented with abdominal pain. CT imaging showed a 2.3- to 2.5-cm SMA aneurysm beyond the origin (Fig 3, *A*). Pathology was negative for infectious or inflammatory etiologies of the aneurysm.

**Fig 4:**
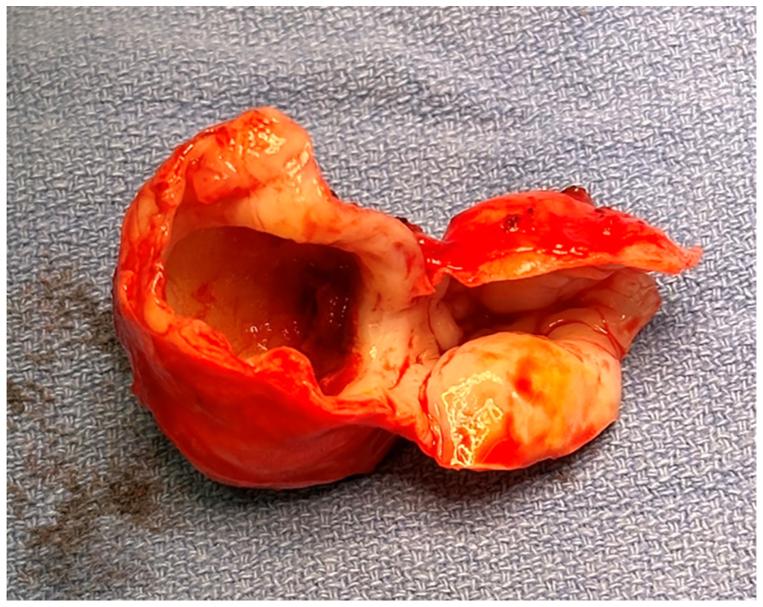
Resection specimen of the superior mesenteric artery (SMA) aneurysm.

**Fig 5:**
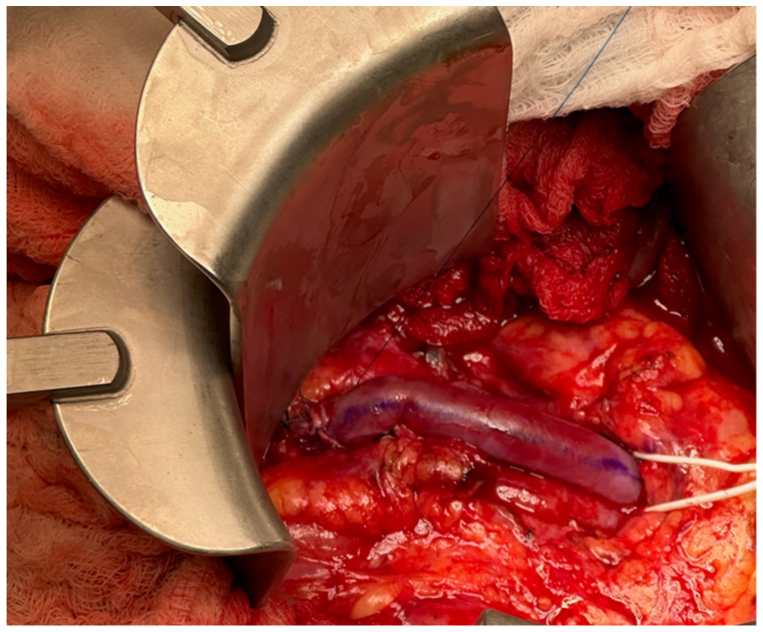
Shows interposition femoral vein graft used for superior mesenteric artery (SMA) reconstruction.

Through a mesenteric approach, the aneurysm was identified, and branches of the superior mesenteric vein were ligated (Fig 3, *B*). The aneurysm was excised (Fig 4), and an interposed femoral-vein graft was sewn using interrupted end-to-end technique (Fig 5). The femoral vein was deemed the appropriate conduit in this situation due to size match needs. Bowel perfusion was brisk on completion; final pathology report demonstrated an SMA aneurysm with focal cystic medial degeneration without inflammation or infection. The postoperative course was uneventful, and there were no signs of any deep venous thrombosis. At 18-month follow-up, the patient remains free of mesenteric symptoms, with a patent bypass on duplex examination. He, however, required a surgical revision of a prolapsed stoma related to his underlying exstrophy. Both patients have provided consent for the publication of this report.

## Discussion

In this report, two cases of VAAs are described in pediatric patients with nonconnective tissue congenital diseases. VAAs are rare conditions not often seen in the general population and are seen far less in pediatric patients.^1^ In the pediatric population, abdominal aortic aneurysms and VAAs are often seen with trauma or other underlying disease processes such as polyarteritis nodosa, other forms of vasculitis, connective tissue disorders, or genetic anomalies.^2^, ^3^, ^4^ A review of the literature shows limited knowledge regarding management of pediatric VAAs, especially in patients with nonconnective tissue conditions such as Down syndrome or cloacal exstrophy. Notably, there has been a report of SMA aneurysms in pediatric patients with PIK3A gene mutations, some in patients as young as 6 weeks old.^2^^,^^5^ Aneurysms of SMA have also been described in association with Alagille syndrome^6^ as a postoperative complication of living donor liver transplantation, and also with no prior transplantation.^7^^,^^8^ It is curious to note that the latter case was found to have a chromosomal mosaicism that was deemed as incidental and unrelated to the patient’s presentation. Alagille syndrome is often characterized due to a loss of function mutation in either the JAG1 or NOTCH pathway.^9^ Despite the variety in congenital mutations and syndromes associated with VAAs in pediatric patients presented in the literature, nearly all of them point to a specific gene; however, mutations in a single gene do not explain the associated congenital anomalies in the two patients described in this case report.

Visceral aneurysms are rare in children, and pediatric-specific management guidelines are lacking. Using adult guidelines, we believed that these aneurysms are at potentially high risk for rupture. In addition, this case report also highlights its association with other syndromes and congenital defects.

Society for Vascular Surgery guidelines for VAAs show that SMA aneurysms should be repaired regardless of size due to potential risk of rupture.^10^ In patients with inflammation, infection, and connective tissue diseases, the risk of rupture is higher and associated with greater morbidity, hence necessitating surgical intervention to prevent fatal outcomes.^11^ Both cases utilized an autologous vein graft as opposed to a synthetic graft, as the autologous conduit offered a higher grade of durability, to reduce the potential risk for infection.^12^ As it applies to any pediatric vascular reconstruction, an interrupted suture technique was used to allow for child growth. Interestingly, the first patient referenced in this manuscript required multiple early angiographic reinterventions, likely due to an inflammatory process. Equally, the need for a stent across the proximal anastomosis was made, as the patient had failed to respond to conventional angioplasty for anastomotic stenosis. Our decision to place a stent will likely pose challenges in the future. We felt this will also allow the child to grow and not require an open surgical revision in the early postoperative period and potential complications. Although internal iliac artery conduits have been used in pediatric vascular reconstructions,^13^ autologous vein conduits provide similar advantages and provide an appropriate size and length match. Pediatric VAAs are unusual with syndromic association and other anomalies. Given their ages, these patients will require lifelong surveillance, similar to any adult patient with VAAs. Guidelines also lack medical therapy recommendations specifically for pediatric arterial aneurysms.

## Funding

None.

## Disclosures

None.
